# CeO_2_:Mn_3_O_4_ Catalytic Micro-Converters Tuned for CH_4_ Detection Based on Catalytic Combustion under Real Operating Conditions

**DOI:** 10.3390/ma13092196

**Published:** 2020-05-11

**Authors:** Cristian E. Simion, Ovidiu G. Florea, Mihaela Florea, Florentina Neaţu, Ştefan Neaţu, Mihaela M. Trandafir, Adelina Stănoiu

**Affiliations:** National Institute of Materials Physics, Atomistilor 405A, 077125 Magurele, Romania; simion@infim.ro (C.E.S.); ovidiu.florea@infim.ro (O.G.F.); mihaela.florea@infim.ro (M.F.); florentina.neatu@infim.ro (F.N.); stefan.neatu@infim.ro (Ş.N.); mihaela.trandafir@infim.ro (M.M.T.)

**Keywords:** CeO_2_:Mn_3_O_4_, CH_4_ detection, catalytic micro-converter

## Abstract

Mesoporous CeO_2_:Mn_3_O_4_ materials (3:7 and 7:3 molar ratio) were prepared by co-precipitation and deposited as porous thick films over alumina (Al_2_O_3_) planar substrate provided with Pt meander. The aim was oriented towards detecting low levels methane (CH_4_) at moderate operating temperatures. Herein we demonstrated that the sensitivity of catalytic micro-converters (CMCs) towards a given peak of CH_4_ concentration corresponds to specific gas-surface interaction phenomena. More precisely, a transition from thermal conductivity to combustion rate is likely to occur when CMCs are operated under real atmospheric conditions (normal pressure, presence of relative humidity, and constant operating temperature). The response to CH_4_ was analyzed over different gas flows and different gas concentrations under the same operating regime. The materials were fully characterized by adsorption-desorption isotherms, H_2_-Temperature Programmed Reduction (H_2_-TPR), X-ray Diffraction (XRD), X-ray photoelectron spectroscopy (XPS), Scanning Electron Microscopy (SEM), and Raman spectroscopies. Thus, the applicative aspect of using CeO_2_:Mn_3_O_4_ as moderate temperature CMC for CH_4_ detection is brought to the fore.

## 1. Introduction

The working principle of a pellistor gas sensor is based on “burning” phenomena. Accordingly, combustion is a chemo-physical interplay between gaseous species capable to combine with the oxygen from the surrounding atmosphere, with heat exchange and secondary products present in the downstream gas. Besides low power consumption, another issue encountered with classical pellistor sensors was related to the sensitive element poisoning, which shortens the sensors’ life [1]. A wide variety of approaches spanning from impregnation with precious metals, 3D highly porous materials, and temperature modulation conditions have been used.

P. Krebs et al. reports pellistor manufacturing onto silicon involving thin film deposition and micromachining techniques [2] which achieve a sensitivity to methane in air of about 13 mV/% CH_4_ when operated at 400 °C. Aigner et al. proposed to operate the pellistor under pulsed temperature modulation (PTM) mode as an alternative to the classic approach [3]. Moreover, Li et al. presented a pellistor system with low energy consumption of up to 30% and a sensitivity of 2.4 mV/% CH_4_. Their approach was based on two-beam microplate technology [4]. A new generation of thermal sensors based on a suspended thermal transistor metal oxide semiconductor (TMOS), fabricated in the standard complementary metal oxide semiconductor—silicon on isulator CMOS-SOI, was recently developed by Nemirovsky et al. [5]. The most important feature of TMOS is given by the high sensitivity exhibited in CH_4_ detection due to the internal amplification of the transistor. Wu et al. [6] reported a novel fabricating process of catalytic gas sensor, which uses a droplet generator based on pulse inertia force to introduce carrier and catalytic materials into a platinum coil in order to fabricate the pellistor. The maximum value of original signal is above 140 mV using a pellistor with the diameter of 450 μm and a catalyst volume of 16 nL. The sensitivity and power consumption of the pellistor are 32 mV/% CH_4_ and 120 mW. Gardner et al. [7] developed a US patent of a so-called micro-combustor. The device is comprised of a micro-hotplate and a catalyst capable to sustain combustion on the microscale.

Among a whole series of metal oxides usable in methane combustion, CeO_2_ (ceria) is one of the most commonly used as heterogeneous catalysts for oxidation reactions [8]. Most of the catalytic properties can be attributed to both the high mobility of the network oxygen and its ease of changing oxidation states from Ce^4+^ to Ce^3+^. The modification of ceria by various metal oxides creates additional structural defect that influences the oxygen storage/release properties responsible for attending good catalytic activity in the methane (CH_4_) combustion. Thus, MnO_x_-CeO_2_ catalytic systems have superior to excellent activity for oxidation of methane at lower ignition temperature, the synergistic effect between the two metal oxides within the solid solution being responsible for this catalytic behavior [9,10,11]. Besides challenges such as high operating temperature and catalyst degradation due to poisoning effects, the shift from using these materials in conventional reactors to preparing gas sensors may involve structural changes that affect the overall performance.

Thus, we recently report that noble metal-free catalysts of Mn- and Ce-based oxides can be used as self-heated catalytic micro-convertors towards detecting methane under real operating conditions (the presence of 50% relative humidity) [12]. Our study indicates that Mn- and Ce-based oxides with reasonable large specific surface area possess good combustion activities that correlate with the strength of the metal-oxygen bond.

Continuing in this research line, herein, we developed a novel interpretation of the gas sensing effects based on the CeO_2_:Mn_3_O_4_ catalytic micro-convertors envisaging the CH_4_ detection. The catalytic results were correlated with morphological and structural aspects of the said materials.

## 2. Materials and Methods 

### 2.1. Materials’ Preparation

All chemicals used in this study for the preparation of catalytic materials came from commercial sources and were used without the need of an additional purification step. The following chemical reagents were used: Cerium (III) nitrate hexahydrate 99.999% (Chemical Abstracts Service (CAS) number 10294-41-4), manganese (II) nitrate hexahydrate 99.99% (CAS number 13446-18-9), and ammonium hydroxide solution (percent concentration of 25%) (CAS number 1336-21-6), all of which were purchased from Merck (Bucharest, Romania). In order to ensure the highest purity of the final materials, Milli-Q deionized water was used in all the preparation steps.

The co-precipitation method was employed in this study to obtain final catalysts with the CeO_2_:Mn_3_O_4_ molar ratio based on oxides of 3:7 and 7:3. The molar ratios were chosen based on our previous research [12] and other studies using Ce-Mn solid solution materials in these ratios for CH_4_ oxidation reaction [13].

The preparation method supposed individual solutions containing the calculated amounts of metal precursors, according to the specified molar ratios to be prepared. Thus, 0.6144 g or 1.6066 g of ceria precursor and 2.4876 g or 1.1946 g of Mn_3_O_4_ precursor were added in 100 mL of Milli-Q deionized water to reach the molar ratios of 3:7 and 7:3, respectively. The solutions were thoroughly mixed (900 rpm) and the pH of the resulting mixtures was adjusted to 10.0 by adding ammonia solution. The mixtures were stirred overnight to ensure maturation of the materials. The resulting solid materials were filtered by using a Buchner funnel and washed with deionized water. Drying of the materials was done under vacuum in an oven at 60 °C for 1 h, then the oven temperature was raised to 160 °C and left overnight. Finally, the materials were calcined at 500 °C for 6 h in air with a heating rate of 5 °C/min.

### 2.2. Materials’ Deposition and Temperature Calibration

**Figure 1:**
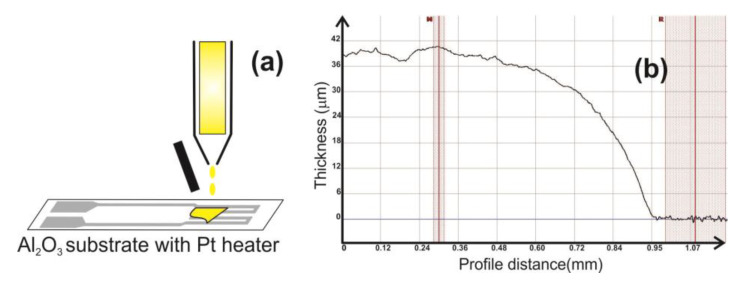
Catalytic materials’ deposition technique onto Pt heater meander (**a**). Film thickness evaluation after drop coating deposition (**b**).

The as-prepared catalytic materials CeO_2_:Mn_3_O_4_ (3:7 and 7:3 molar ratio) were mixed with an organic binder (1,2-propanediol) and further deposited onto platinum heater meander of Al_2_O_3_ planar substrates via drop coating technique (Figure 1a). Few drops hanging on a capillary were gently attached to the planar Al_2_O_3_ surface. Due to the surface tension of the paste, deposition had a limited spreading by the edges of the substrate. The transferred sensitive paste was dried at 60 °C for 18 h followed by a steep increase in temperature up to 400 °C (50 °C at 30 min). At the final firing temperature of 450 °C, the catalytic micro-convertors were kept for 1 h for better stabilization of the morphological and structural architecture. The thickness of the CeO_2_:Mn_3_O_4_ layer was around 40 µm (Figure 1b). The thickness measurements were performed using an XP-Plus Stylus Profilometer (XP200, AMBIOS, New York, NY, USA) operated at room temperature under laboratory conditions.

**Figure 2:**
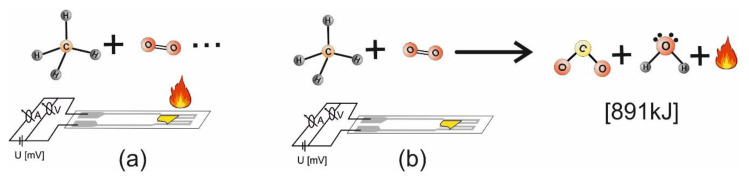
Pt heater meander ensuring gas catalysis (**a**) and combustion temperature evaluation (**b**).

The Pt heater meander (as depicted from Figure 2) had two functions, one was responsible for heating the catalytic materials (a) while the second was responsible for temperature evaluation of the subsequent combustion processes (b).

Planar Al_2_O_3_ substrates (96% purity and 700 µm thickness) were chosen as standard solution for our electrical setup used for the evaluation of the catalytic performances of CeO_2_:Mn_3_O_4_ (3:7 and 7:3 molar ratio). Moreover, since these planar substrates can be directly plugged into a PTFE (polytetrafluoroethylene) connector of the micro-convertors’ chamber through its length of 25.4 mm, the thermal stress and dissipation was considerably minimized. This was our prerequisite to be able to compare the performances of two different materials and to explain their catalytic behavior observed.

Prior to gas sensing investigations, the micro-convertors were subjected to temperature calibration. Such catalytic materials are usually operated at elevated temperatures 200 °C and 600 °C. As a way to maintain the temperature constant during the operation, it was necessary to ensure a voltage drop over the Pt heater meander.

**Figure 3:**
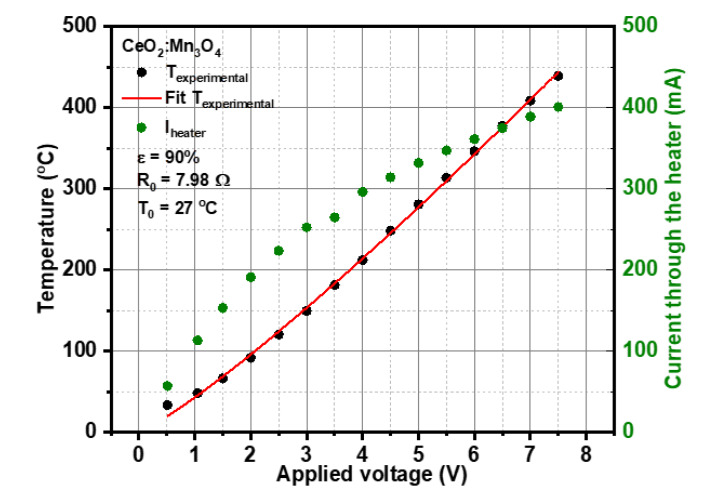
The calibration points and their fitting curve spanning from 50 °C to 400 °C.

In order to establish a relationship between the attained temperature with respect to the applied voltage and current through the heater, a subsequent calibration procedure was applied. The resulting calibration curve, details about specific emission coefficient ε of the material, and electrical resistance at room temperature are presented in Figure 3. The applied calibration law extracted through the fitting curve was $U^{1.16}=T/k$, where *U* represents the applied voltage to the Pt heater meander and *k* is a material constant (*k* = 42.79) and *T* is the operating temperature.

**Figure 4:**
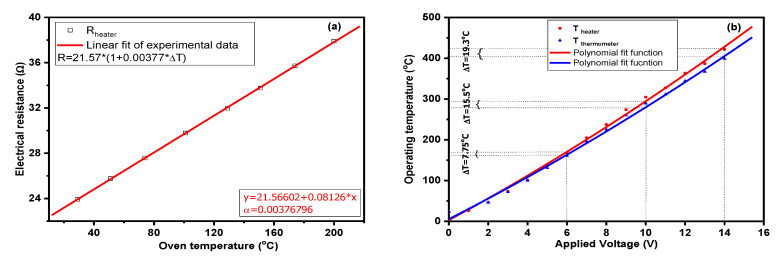
Pt heater temperature coefficient determination (**a**), operating temperature with respect to the applied voltage (**b**).

Knowing the importance of the operating temperature of the catalytic micro-convertors and the way in which the setting temperature by the Pt heater is influenced by various intrinsic (e.g., Al_2_O_3_ temperature dissipation) or extrinsic (e.g., surrounding gas atmosphere) factors, the temperature coefficient of the Pt heater was found from the electrical resistance measurements using a thermostat oven (Figure 4a).

It can be observed in the plot above (Figure 4b) that a maximum temperature difference of approximately 20 °C existed due to the heat transmission of the alumina substrate from one face to the other. A second order polynomial fit was performed to comprise data corresponding to temperatures below 100 °C. For such evaluation a double Pt heater, bare Al_2_O_3_, was used, i.e., face-to-face heater separated by the alumina substrate.

The pellistors were tested under constant heater voltage operating mode. In this situation the Pt heater was connected to a voltage source and the current was measured. Usually the thermal stability is achieved in time of seconds. The major issue is related only to the case when the catalytic combustion is of the same order of magnitude as the electrical heating power because this will be translated into an overheated element. Such behavior was not recorded in the presented measurements.

### 2.3. Materials’ Characterization

To obtain information about the properties of as-prepared samples, several characterization techniques were employed in this study. Thus, the vibrational properties were obtained by Raman spectroscopy. The morphological and structural properties, crystallinity of the samples, and the type of crystalline phases present in the material were verified by scanning electron microscopy (SEM), X-ray photoelectron spectroscopy (XPS), and powder X-ray diffraction (XRD). Determination of the degree of reduction and reducibility of the active elements present in the materials was achieved by temperature programmed reduction (TPR) experiments, while the textural properties were determined by nitrogen adsorption–desorption isotherms at the nominal temperature of liquid nitrogen and at a wide relative pressure range from 0.01 to 0.995.

Raman measurements were performed in the range between 100 and 1200 cm^−1^ on a LabRAM HR evolution spectrometer (Horiba Jobin Yvon, Kyoto, Japan) equipped with an air-cooled Charge Coupled Device (CCD) and a He-Ne laser (633 nm). All the Raman spectra were recorded at room temperature in the extended scan mode with an acquisition time of 5 × 50 s.

A GeminiSEM 500 microscope (Carl Zeiss AG, Oberkochen, Germany) equipped with a Bruker EDX (Energy-dispersive X-ray spectroscopy) Quantax XFlash spectrometry detector (Quantax XFlash, Bruker, Karlsruhe, Germany) for elemental analysis was used to perform the SEM measurements. Images of all samples were taken at the same magnification of 10,000× and acceleration voltage of 1 kV using the InLens detector (Carl Zeiss AG, Oberkochen, Germany).

XPS measurements were realized on a Kratos Ultra DLD Setup spectrometer (Kratos Aalytical Ltd., Manchester, UK) using the Al–Kα (1486.74 eV) radiation produced by an X-ray source operating at a total power of 300 W (12.0 kV × 25 mA) and an approx. 1 × 10^−7^ Pa.

The X-ray diffraction measurements were performed using a Bruker-AXS D8 Advance diffractometer (Bruker Corporation, Billerica, MA, USA.) equipped with a LynxEye 1D detector and Cu-Kα (0.1541 nm) radiation source and a scintillation counter detector. The diffraction patterns were recorded over a 2θ range of 10°–80° with a 0.01° step size and using a counting time of 1 s per point. The identification of the XRD phases present in the samples was realized by using the Powder Diffraction File from the International Centre for Diffraction Data (PDF-ICDD). Particle size (D) of nanocrystalline ceria was calculated using Scherrer formula where coefficient of anisotropy was set to 0.9. D values were calculated from full width at half maximum (FWHM) for the most intense diffraction lines: (111) for CeO_2_ and (101) for Mn_3_O_4_.

The temperature programmed reduction experiments in hydrogen atmosphere (H_2_–TPR) were realized by using a Porotec TPDRO 1100 device (Thermo Fisher Scientific Inc., Waltham, MA, USA). Prior to the reduction step, approximately 50 mg of sample was pretreated for 1 h at 200 °C in a helium gas flow to ensure the surface cleaning, after which it was cooled down to room temperature, also in a helium gas flow. Then a 5 vol.% H_2_–Ar mixture was passed over the sample with a flow rate of 50 mL/min and the temperature was linearly increased by 10 °C/min to 800 °C. Using the equipped thermal conductivity detector of the TPDRO device, we carried out the quantification of the hydrogen consumption during the reduction process.

The surface areas and pore size distribution of the as-prepared materials were determined by N_2_ adsorption–desorption isotherms at liquid N_2_ temperature (−196 °C) on a Micromeritics (ASAP 2020) analyzer (Micromeritics Instrument Corporation, Norcross, GA, USA). Specific surface area and pore size distribution were calculated by Brunauer–Emmett–Teller (BET) formalism [14] and Barrett–Joyner–Halenda (BJH) method [15], respectively. In the case of all samples, in order to efficiently remove the surface adsorbed residues, a degassing step at 150 °C for 4 h was employed.

### 2.4. Electrical Characterization

**Figure 5:**
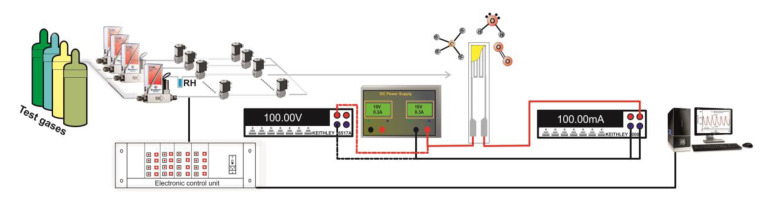
Electrical setup used for CeO_2_:Mn_3_O_4_ (7:3 or 3:7) catalytic micro-convertors characterization.

The catalytic micro-convertors were tested towards CH_4_ detection under constant and variable gas flow using a fully computer-controlled gas mixing system, described elsewhere [12]. The current passing through the Pt heater meander was measured by means of a Keithley 2000 A multimeter while the applied voltage was recorded using a Keithley 6517 A electrometer. Prior to each investigation, the catalytic micro-convertors were installed in a sealed PTFE chamber and exposed to normal atmospheric conditions for 1 h (constant gas flow, synthetic air with 50% relative humidity (RH)). The synthetic air (20% O_2_ and 80% N_2_) and CH_4_ diluted in synthetic air, used in the experimental measurements, were provided from certified high purity gas cylinders (Figure 5).

Such preconditioning procedure was necessary in order to acquire a stable electrical current baseline and to avoid further drifts’ issues during the first use of the sensitive CeO_2_:Mn_3_O_4_.

In order to examine the differences between the molar aspect ratio between the catalytic micro-convertors, systematic investigations were performed towards 2500 ppm CH_4_ for operating temperatures between 25 °C and 400 °C. After finding the peculiar characteristic behavior for each catalytic material, the materials were subject to various gas flow levels’ investigation as a way to highlight the differences induced by the presence of different porous systems. Last gas sensing investigations were oriented to address a possible sensing mechanism using CeO_2_:Mn_3_O_4_ catalytic micro-convertors by spanning the electrical response over a wide range of CH_4_ concentration at fixed gas flow level and operating temperature.

## 3. Results

### 3.1. Materials’ Characterization

#### 3.1.1. Raman Spectroscopy

**Figure 6:**
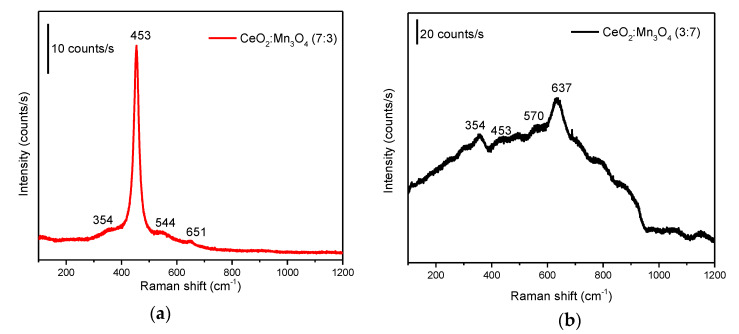
The Raman spectra of the CeO_2_:Mn_3_O_4_ materials with the molar ratios (**a**) 7:3 and (**b**) 3:7.

To gain more information about the structure of the CeO_2_:Mn_3_O_4_ samples, Raman spectra recorded at room temperature are shown in Figure 6. Raman spectroscopy is seen as a powerful characterization technique for screening the nature of the active species from the catalyst’s surface. Here we could include the O-vacancies and the defect sites that have a great influence over the surface reactivity by modifying the local electronic structure of the catalyst [16,17,18,19]. In this aspect, we can expect that, depending on the molar ratio used in the synthesis, the CeO_2_:Mn_3_O_4_ materials to possess different active sites that influence the overall catalytic activity.

For instance, as shown in Figure 6a for CeO_2_:Mn_3_O_4_ (7:3) sample, the strong Raman band centered at around 453 cm^−1^ can be safely associated to F2g mode of fluorite CeO_2_ [20,21]. It has to be pointed out that the Raman characteristic band for pure CeO_2_ appeared at ca. 465 cm^−1^, but, by doping with Mn species, a significant shift to lower frequency at 453-460 cm^−1^ took place and, thus, this red-shift of F2g band can be assigned to the formation of O-vacancies. Moreover, that suggests the formation Ce–O–Mn solid solution. The presence of separated phases of Mn_3_O_4_ in this case (see Figure 6a) can be foreseen through the appearance of two broad bands located at 651 cm^−1^ and 354 cm^−1^, bands that can be assigned to the Mn–O–Mn stretching and bending modes, respectively [22,23]. Actually, the presence of these bands indicated that the incorporation of manganese in CeO_2_ led also to the formation of a solid solution to a segregation of Mn_3_O_4_ phase, an observation that is in good agreement with the XRD results.

Apart from these bands, the Raman spectrum of CeO_2_:Mn_3_O_4_ (7:3) showed a shoulder band at around 544 cm^−1^ (see Figure 6a), which can be ascribed as the defect-induced mode band, which is sensitive to the formation of O-vacancies [24].

When comparing the Raman spectra between CeO_2_:Mn_3_O_4_ (7:3) and CeO_2_:Mn_3_O_4_ (3:7), aside from the CeO_2_ F2g band at 453 cm^−1^, a red-shift to 637 cm^−1^ of the band assigned to the Mn–O–Mn stretching mode was observed (see Figure 6b), while the band located at 354 cm^−1^ and assigned to the Mn–O–Mn bending mode of Mn_3_O_4_ phase remained unchanged. Such red-shift can be the indication of the presence/formation of a new, separated MnO_2_ phase [25]. These results were, once again, in good agreement with the XRD results, presented further.

#### 3.1.2. X-ray Diffraction Analysis

**Figure 7:**
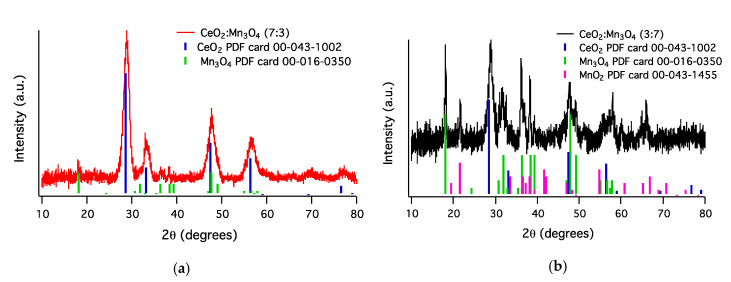
XRD patterns of the CeO_2_:Mn_3_O_4_ (7:3) (**a**) and (3:7) (**b**) together with the PDF cards of CeO_2_ (00-016-0350) Mn_3_O_4_ (00-043-1002) and MnO_2_ (00-043-1455) assignments.

The XRD patterns of the two materials are displayed in Figure 7. For the sample CeO_2_:Mn_3_O_4_ (7:3) containing a high amount of Ce, preponderantly CeO_2_ phase assigned to cubic fluorite structure (Powder Diffraction File (PDF)card 00-043-1002) can be observed, while only low intensity diffraction lines can be associated with the orthorhombic Mn_3_O_4_ phase (PDF card 00-016-0350) (Figure 7a). A closer look at the lattice parameter of CeO_2_ fluorite cubic structure shows a decrease of a_0_ parameter, for instance, from 0.541 nm for pure ceria [26] to 0.536 nm for CeO_2_:Mn_3_O_4_ (7:3) sample (Table 1). This behavior indicates the formation of a solid solution containing Ce and Mn, when Mn^3+^ replaces Ce^4+^ in the fluorite cubic structure, and is due to the lower ionic radius of Mn^3+^ (0.66 nm) compared to Ce^4+^ (0.94 nm). The Ce^4+^ replacement proceeded very easily due to the structural similarities between the two elements; behavior also observed by others [27,28]. The sample CeO_2_:Mn_3_O_4_ (3:7) enclosing a high amount of Mn had a different distribution of phases than the sample with lower amount of Mn. Thus, CeO_2_ cubic fluorite structure (PDF card 00-043-1002) and orthorhombic Mn_3_O_4_ phase (PDF card 00-016-0350) were also identified, with the amendment that the diffraction lines of Mn_3_O_4_ phase were more intense for this sample (see Figure 7b). Also, the presence of MnO_2_ orthorhombic phase (PDF card 00-012-0715) crystalized in small amounts should be taken into consideration for this sample. This phase was evidenced also by Raman spectroscopy. The CeO_2_ cubic fluorite in this case had a smaller lattice a_0_ parameter than the pure ceria (Table 1) and even the CeO_2_:Mn_3_O_4_ (7:3) sample, meaning that also in this case the Mn^3+^ replaced partially the Ce^4+^, forming a solid solution between the two oxides. For both samples, very small crystallites’ size for the CeO_x_-MnO_x_ solid solution formed (~7 nm) can be observed, while the manganese oxides presented bigger crystallites (~30 nm) (see Table 1).

#### 3.1.3. X-ray Photoelectron Spectroscopy

**Figure 8:**
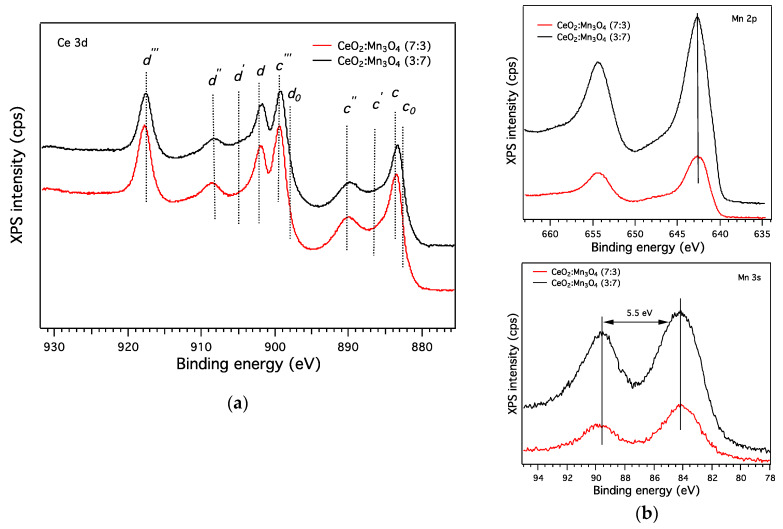
The XP spectra of the samples CeO_2_:Mn_3_O_4_ (7:3) and (3:7) in the Ce 3d (**a**), Mn 2p, and Mn 3s (**b**) regions.

XPS was used to determine the components found at the surface of the two materials. A correction at C 1s at 284.6 eV was used to calibrate the spectra; and from the wide spectra, Mn, Ce, C, or O were identified in the samples. The high-resolution spectra of Ce 3d (Figure 8a) showed only three doublets (c/d, c’’/d’’, c’’’/d’’’) from the five doublets possible (c_0_/d_0_, c/d, c’/d’, c’’/d’’, c’’’/d’’’). The three doublets identified, in both samples, belonged to +4 oxidation state, as found in XRD and H_2_-TPR data. The doublets c_0_/d_0_ and c’/d’ attributed to the oxidation state Ce^3+^ could not be assigned. The high-resolution spectra Mn 2p and Mn 3s were recorded (Figure 8b). Mn 2p presented two doublets, Mn 2p_3/2_ and Mn 2p_1/2,_ found at 642.7 eV and 654.3 eV, respectively with a split energy of 11.6 eV, which corresponded to Mn_3_O_4,_ according to other studies [29,30]. Moreover, the splitting Mn 3s spectra indicated an energy of 5.5 eV, assigned also to Mn_3_O_4_ [31], confirming that at the surface of both samples Mn_3_O_4_ was present. The Ce/Mn ratio found at the surface by XPS analyses showed the same trend as in bulk (Table 1). However, the difference in values found can be explained by the fact that XPS is a surface analysis technique and can detect only max. 10 nm from the surface.

#### 3.1.4. Temperature Programmed Reduction Analysis

**Figure 9:**
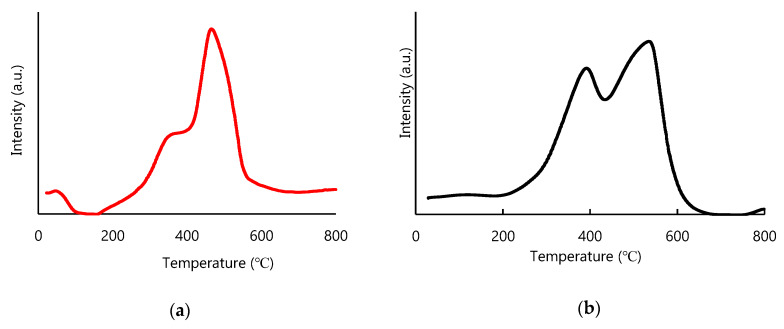
H_2_-TPR profiles of the CeO_2_:Mn_3_O_4_ (7:3) (**a**) and (3:7) (**b**).

H_2_-TPR experiments were performed in order to gain more information about the redox properties of the synthesized samples (see Figure 9).

As can be observed, both samples exhibited relatively similar behavior under reduction atmosphere, with two reduction peaks appearing in each case, named low and high temperature peak, respectively. The intensity of these peaks was proportional to the amount of CeO_2_ and Mn_3_O_4_ found in the samples. The first reduction peak below 400 °C, the low temperature one, was ascribed to the Mn^4+^/Mn^3+^(Mn^2+^) reduction process, which was found in the curve of CeO_2_:Mn_3_O_4_ (7:3) at around 360 °C (Figure 9a) and at around 393 °C for the CeO_2_:Mn_3_O_4_ (3:7) sample (Figure 9b) [28]. The difference between the reduction temperatures indicates a higher reducibility degree and, consequently, higher oxygen mobility for the sample with higher amounts of CeO_2_. Also, this shift to lower temperature could be due to the interaction between manganese and cerium oxides with formation of solid solution in which the mobility of oxygen species is much higher [32]. This correlates well with Raman and XRD techniques, which evidenced, as well, the formation of a solid solution. With the increase of the temperature above 400 °C, the H_2_-TPR profiles showed a second peak ascribed to the reduction of the surface CeO_2_ trough, a Ce^4+^ to Ce^3+^ reduction process. This occurred at 466 °C and 540 °C in the case of CeO_2_:Mn_3_O_4_ with the molar ratio of 7:3 and 3:7, respectively [33]. However, the contribution of the reduction of Mn_3_O_4_ to MnO for the high temperature peak cannot be excluded [26].

As expected, the ratio between the low to the high temperature reduction peaks was much higher for the sample CeO_2_:Mn_3_O_4_ (3:7), which contained more manganese oxide species with higher oxidation states.

#### 3.1.5. Textural Characterization

**Figure 10:**
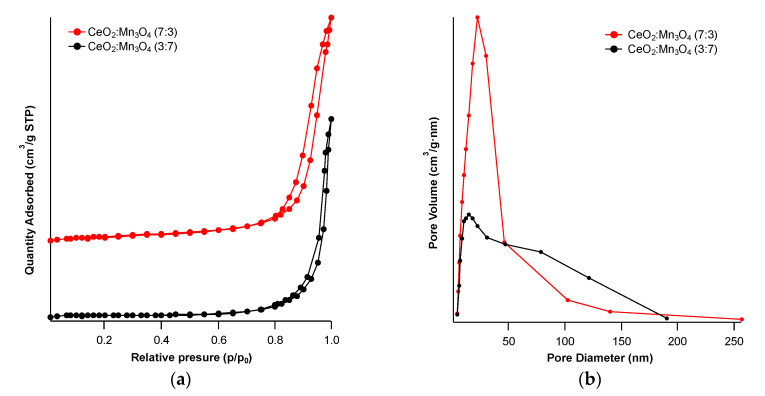
Nitrogen adsorption-desorption isotherms (**a**) and pore size distribution (**b**) of the two oxides CeO_2_:Mn_3_O_4_ (7:3) and CeO_2_:Mn_3_O_4_ (3:7).

The surface area of both prepared materials was calculated using BET formalism from the nitrogen adsorption-desorption isotherms (Figure 10).

According to the shapes, the nitrogen desorption occurred at lower relative pressures than the adsorption, giving rise to a IV type isotherm [33], specific to mesoporous materials (see Figure 10a).

A H3 hysteresis loop characterized both materials, since nitrogen desorption was different than adsorption due to the capillary condensation, which happened in the mesopores. This kind of hysteresis is associated with slit-shaped meso- and/or macropores. This was confirmed also by the pore size distribution (Figure 10b), where, for the sample containing a high amount of Ce, only mesopores were present in a window of 0–50 nm, while the sample containing mainly manganese both kinds of pores were present (meso- and macropores).

The surface area decreased with the increase of manganese content (Table 2) from 52 m^2^·g^−1^ to 29 m^2^·g^−1^. This behavior was also observed in other studies [30,34] and was due to the grain growth when the amount of manganese oxide was increasing in the sample, growth observed also by XRD analysis in the CeO_2_:Mn_3_O_4_ (3:7) sample. Indeed, the calculated average grain size for the CeO_2_:Mn_3_O_4_ (3:7) sample was bigger than that of CeO_2_:Mn_3_O_4_ (7:3) (last column in Table 2), which was in agreement also with the crystallite size measured using XRD data (see Table 1).

#### 3.1.6. Scanning Electron Microscopy

**Figure 11:**
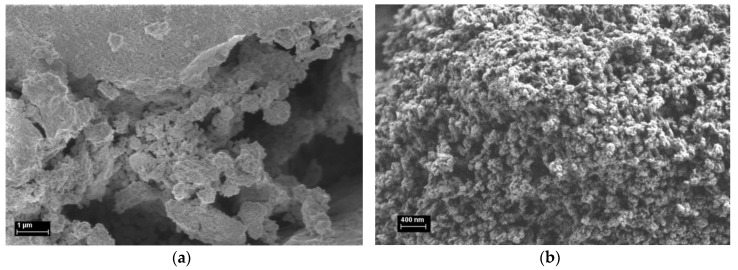
SEM images at 10,000× and 20,000× magnification of the CeO_2_:Mn_3_O_4_ materials prepared by co-precipitation method with the following molar ratios: (**a**) 7:3 and (**b**) 3:7, respectively.

Images of the as-prepared samples are presented in Figure 11 and, for comparison, were taken at magnifications of 10,000× and 20,000× and acceleration voltage of 1 kV using the InLens detector.

SEM images of the CeO_2_:Mn_3_O_4_ materials showed that the samples were agglomerated but presented fine pores on the surface. Actually, the sample with a higher amount of Mn_3_O_4_ (Figure 11b) apparently showed much bigger pores than the CeO_2_:Mn_3_O_4_ (7:3) sample (Figure 11a). This observation was further confirmed by the nitrogen adsorption-desorption experiments, which showed that the pore diameters of the CeO_2_:Mn_3_O_4_ (3:7) sample were almost double in size than that of CeO_2_:Mn_3_O_4_ (7:3) (see Section 3.1.5).

### 3.2. CH_4_ Catalytic Combustion Results

In order to gain insights about fundamental and applicative properties of CeO_2_:Mn_3_O_4_ and catalytic micro-convertors, several investigations were addressed.

**Figure 12:**
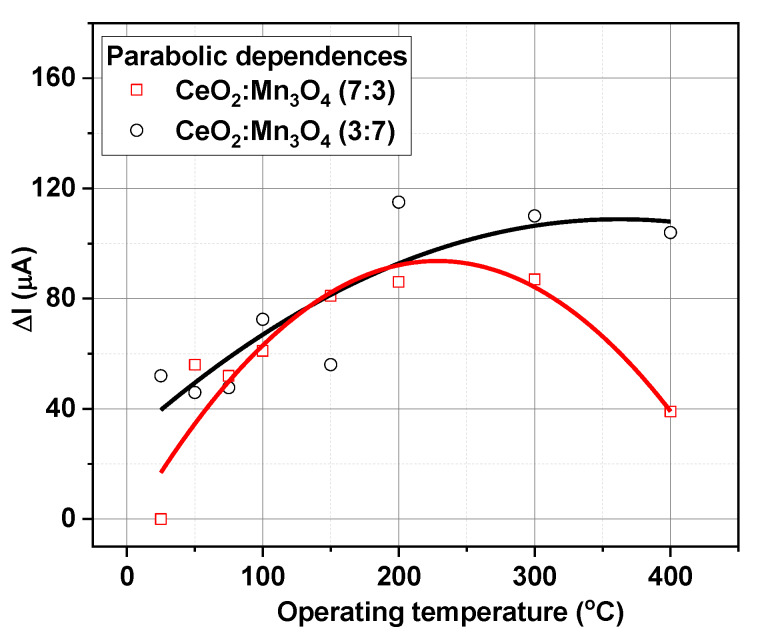
Electrical current difference Δ*I* upon 2500 ppm CH_4_ exposure under 50% relative humidity at different operating temperatures.

The optimal operating temperature towards detection of 2500 ppm CH_4_ under 50% RH was investigated by testing the catalytic materials CeO_2_:Mn_3_O_4_ (3:7 and 7:3) at different temperatures (from 25 to 400 °C). The aforementioned gas concentration was chosen as half from the lower explosive limit (LEL). Accordingly, gas concentrations lower than the LEL were ‘too lean’ to burn, whereas those above this limit were highly explosive up to a level when they became too rich to burn. Figure 12 shows the variations of the electrical current passing through the heater, in terms of $\left|\Delta I\right|=I_{air}-I_{gas}$, were |ΔI| represents the difference in the current passing through the heater; $I_{air}$ is the electrical current measured under the presence of air with 50% RH and $I_{gas}$ is the electrical current measured in the presence of CH_4_, following a well-defined gas sensing protocol, namely: At each operating temperature the catalytic micro-convertors were kept for 1 h under constant flow of synthetic air with 50% RH for stabilization, followed by 15 min exposure to 2500 ppm of CH_4_. After each gas exposure the recovery times was set to 30 min.

As can be seen in Figure 12, the difference in the current passing through the heater showed a parabolic dependence with different maxima with respect to the catalytic material. Thus, in the case of CeO_2_:Mn_3_O_4_ (3:7) micro catalytic-convertors, the evolution of the electrical current was fitted with a half of a parabolic arch, while in the case of CeO_2_:Mn_3_O_4_ (7:3) a complete parabola was obtained with respect to the operating temperatures.

The aforementioned behaviors could be related to the difference that occurred in both porosity and molar ratio between the two oxides. Hence, one can see that in the case of CeO_2_:Mn_3_O_4_ (3:7) there was a necessary temperature of about 400 °C. In order to ensure CH_4_ combustion for the case of CeO_2_:Mn_3_O_4_ (7:3), the maximum variation in the current passing through the heater occurred at 223 °C (as computed from the hyperbolic fit parameters). Such behavior is typical for most of the flammable gas sensors based on catalytic measurements. It should be noted that CeO_2_:Mn_3_O_4_ (7:3) had oxygen vacancies and exhibited a high degree of reducibility (the ability to lose lattice oxygen), which made it more active in interaction with CH_4_.

While, for the case of the micro-convertor with the molar ratio of 3:7, the possible detection mechanism envisaged a simple catalytic conversion process and the later exhibited a more interesting behavior. Accordingly, the volcano-like shape in the case of CeO_2_:Mn_3_O_4_ (7:3) can be explained based on the following relationship:

(1) $$\Delta I=k\times \Delta H\times Dif\times c_{CH4}$$

where ΔI is the difference in the electrical current passing through the heater, k is a material constant, ΔH is the difference in the caloric power of CH_4_, Dif represents the diffusion coefficient through the porous catalyst, and $c_{CH4}$ is the CH_4_ concentration [35].

On the ascendant trend, the increase in the electrical current passing through the heater could have been related to the catalytic function exhibited by CeO_2_. Ceria is known to be able to pick up and store oxygen from the gas phase under fuel-lean operating conditions (excess oxygen) and to release it under fuel-rich conditions (excess fuel) for reaction with CH_4_. Thus, it effectively *dampened* the variations in the air/CH_4_ ratio as the exhaust gas mixture cycled, thus helping to maintain the full operation within the desired window for optimum conversion over the catalyst material.

On the descendent trend, the decrease in the electrical current difference could be possibly justified by fast combustion of the CH_4_ over the heated surface of catalyst, leading to less diffusion phenomena inside the catalyst bulk, thus lessening conversion of CH_4_ into downstream gas products.

In the framework of all the above-mentioned results, we decided that the subsequent catalytic measurements would be performed at 223 °C as maximum variation in the current passing through the heater.

Most of the gas sensing concepts include either static or constant gas flow measurements of the subsequent detection principles. Accordingly, in order to investigate the role of gas flow over the catalytic properties of CeO_2_:Mn_3_O_4_ (3:7 and 7:3), the gas sensors were mounted into a chamber where the gas flow was free of turbulence.

**Figure 13:**
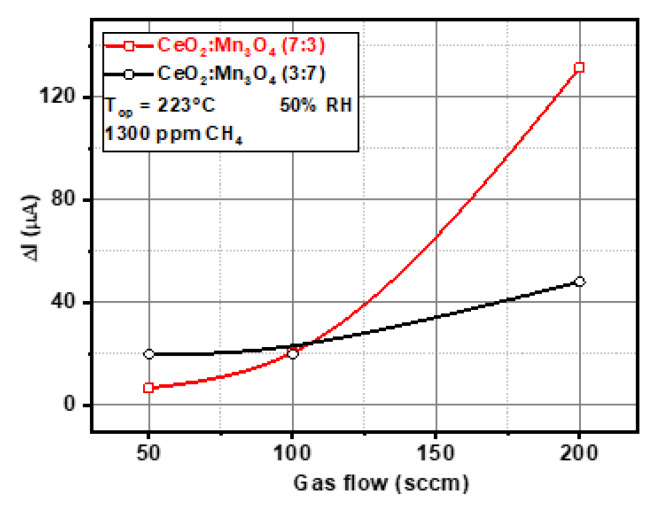
Differences occurred in the current passing through the heater as computed for CH_4_ exposure using different gas flows.

Thus, the catalytic micro-convertors were exposed to the same gas testing protocol repeated for each gas flow rate (50, 100, and 200 mL/min). The stabilization period was of 30 min, followed by 1300 ppm CH_4_ in air with 50% RH, with a recovery time of 15 min. The calculated differences within the current passing through the heater for both catalytic materials revealed the way in which the intrinsic properties of the material were reflected over their catalytic behavior (see Figure 13).

In both cases, the catalytic properties of CeO_2_:Mn_3_O_4_ (3:7 and 7:3) were acquired by measuring the current difference between reference atmosphere (air with 50% RH) and gas atmosphere (air with 50% RH and 1300 ppm CH_4_) under constant voltage operating mode. The effect seen in the electrical current difference with respect to the catalytic materials can be attributed to the textural properties, namely CeO_2_:Mn_3_O_4_ (3:7 and 7:3), which exhibited a ratio of (29 and 52) in terms surface area. The higher the porous structure was then the higher the influence of air convection through the layer was (see Table 2).

**Figure 14:**
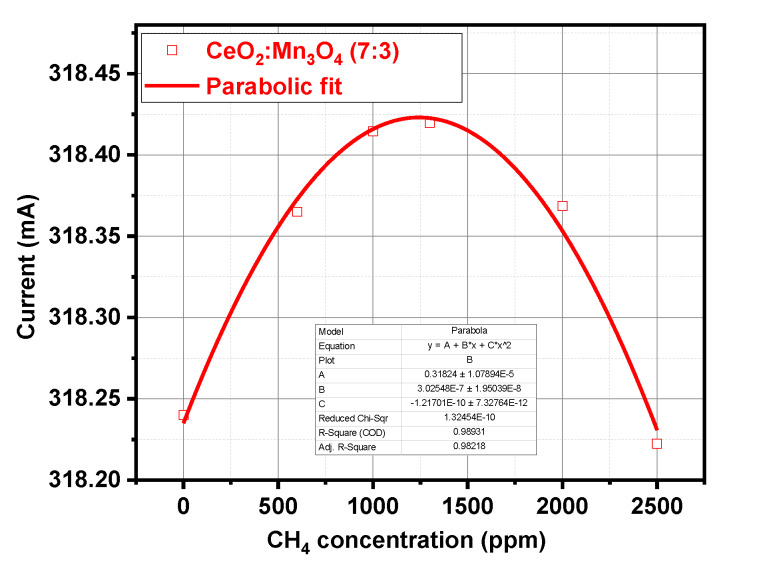
The electrical current passing through the heater, dependence with respect to the CH_4_ concentration for CeO_2_:Mn_3_O_4_ (7:3).

The use of porous catalytic structures had the advantage of increasing the overall response to CH_4_ against its strong dependence on the upstream gas flow level. The diffusion occurred as a result of the tendency of the gases to occupy all the available volume, due to the thermal agitation. This process led to the equalization of the concentration differences (at constant Pressure and Temperature) as an expression of the natural tendency of the systems to reach the equilibrium state. Fick’s laws characterize the diffusion phenomenon and it has a nonlinear dependence for systems with different porosity. It is easy to understand that the aforementioned difference was given by the gas concentration gradient inside the pores, according with the Knudsen diffusion relation [36]. In this case, the mesoporous structure was responsible for the volcano-like shape described in Figure 14, just for the case of CeO_2_:Mn_3_O_4_ (7:3). However, this interesting behavior can be explained by the fact that this sample presented a better reducibility as observed from H_2_-TPR experiments and also due to the presence of O-vacancy, as indicated by Raman spectroscopy. Increasing the amount of manganese was detrimental to such properties and, as a consequence, the catalytic behavior diminished.

## 4. Discussion

From the presented results, we noted that micro-catalyst CeO_2_:Mn_3_O_4_ (7:3) had a peculiar behavior towards CH_4_ detection and so we decided to pursue further investigations of its catalytic behavior.

In order to highlight the possible gas-surface interaction mechanisms of the CeO_2_:Mn_3_O_4_ (7:3) micro-catalytic convertors, this was exposed to different CH_4_ gas concentrations (600, 1000, 1300, 2000, and 2500 ppm) under constant gas flow (200 sccm), constant temperature (223 °C), and constant relative humidity (50% RH). The as-selected micro-convertor operated on the principles of heat detection, further transformed into a measured physical unit (i.e., electrical current). It is known that the total oxidation of methane released around 803 kJ/mol, [37] while in the presence of 50% RH the heat was even higher, according to the Equation (2).

(2) $${\mathrm{CH}}_{4}+2O_{2}+8N_{2}\overset{50\%\mathrm{RH}}{\to }{\mathrm{CO}}_{2}+2H_{2}O+8N_{2}+\Delta_{H}\left[891\mathrm{kJ}\right]$$

As one can see in Figure 14, the CeO_2_:Mn_3_O_4_ (7:3) micro-convertor showed a parabolic dependence with respect to the CH_4_ concentration. This behavior was first reported by J. Chou [38] in the case of methane detection using pellistor-type sensors.

**Figure 15:**
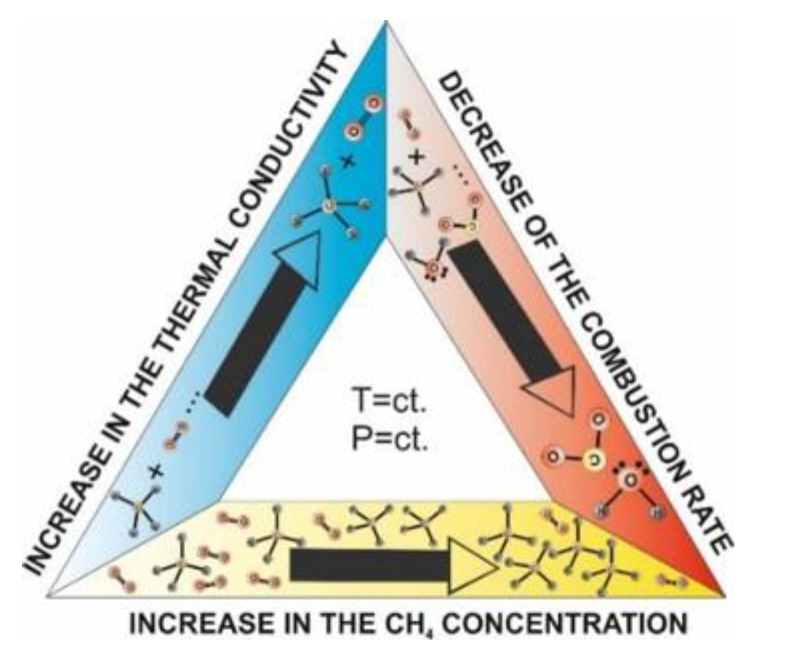
Schematic drawing of the involved processes within the catalytic behavior with CeO_2_:Mn_3_O_4_ (7:3).

In order to explain the electrical current trend in the ascending branch (0–1300 ppm of CH_4_), one has to consider the thermal transfer from the catalyst to the surrounding atmosphere (Figure 15). Such effect is possible knowing that the thermal conductivity of methane is up to 40% higher in comparison with air [39]. Accordingly, one can notice that the increase in the electrical current, i.e., the decrease in the electrical resistance of the Pt heater meander, is a consequence of the global “cooling” phenomena with several tenths of degree Celsius. This might be translated that the fuel: air mixture is too “lean”.

Once the ratio CH_4_:O_2_:N_2_:RH attained the critical point, the combustion rate started to decrease with the increase in CH_4_ concentration. This might be translated that the fuel:air mixture is too “rich”.

On the other hand, in the presence of a good catalyst, the target gas will begin to ignite at a lower operating temperature [40] than usual. It is worth mentioning that for detection of gases with high thermal conductivity (usually greater than air), such as methane, the detection principle based on temperature difference is working [41]. Higher power pellistors have greater catalytic activity and are less vulnerable to poisoning. More porous beads also have greater catalytic activity as their surface increases [6].

Putting all this together, we can say that an inversion effect from thermal transfer to catalytic conversion takes place at a threshold value of 1300 ppm of CH_4_. The overall combustion of CH_4_ is mirrored by a difference in the electrical current of 20 μA. With regard to potential interferences, no visible effects were seen for H_2_S from the preliminary selective sensitivity measurements. Cross-sensitivity to other potential interfering gases and possible contamination are concerns for the near future beside technological development of micro-coils’ low power consumption applications.

## 5. Conclusions

Mesoporous mixed oxides containing different Ce/Mn ratios were successfully prepared by a simple and cost-efficient co-precipitation method. The formation of a solid solution was evidenced for both CeO_2_:Mn_3_O_4_ (3:7) and CeO_2_:Mn_3_O_4_ (7:3) samples by different characterization techniques; however, to a lesser extent for CeO_2_:Mn_3_O_4_ (3:7), which presented also segregated manganese oxides phases. We identified several factors to be responsible for the improvement of the catalytic behavior of CeO_2_:Mn_3_O_4_ (7:3) sample: (1) the surface area and the pore volume, (2) the formation of oxygen vacancies, and (3) higher reducibility. We could address a possible reaction mechanism aiming to explain the parabolic catalytic behavior of the CeO_2_:Mn_3_O_4_ (7:3) CMC when operated under real conditions. Gas sensing experiments undertaken in this paper had the objective to detect CH_4_ under real operating conditions. Thus, we were able to detect traces of CH_4_ in the range 600 ppm to 2500 ppm using inspired mixed oxides. As before depicted, the detection of CH_4_ at low operating temperature and under the presence of moisture was a quite challenging perspective. Therefore, the future applicative direction is to use micro-coils coated with CeO_2_:Mn_3_O_4_ capable of fast heating rates in order to trace rapidly the thermal signature of different concentrations of CH_4_.

## Figures and Tables

**Table 1:** Crystallite size, lattice parameters, and Ce/Mn ratios.

Sample	Composition	Ce/Mn Molar Ratio		Lattice Parameters	Crystallite Size (nm)
		(Ce/Mn) ^a^	(Ce/Mn) ^b^	a_0_ (nm)	
CeO_2_:Mn_3_O_4_ (7:3)	CeO_2_	2.33	0.23	0.536	6
	Mn_3_O_4_				27
CeO_2_:Mn_3_O_4_ (3:7)	CeO_2_	0.43	0.11	0.533	7
	Mn_3_O_4_				33
	MnO_2_				10

^a^ In bulk, ^b^ from XPS.

**Table 2:** Textural properties of CeO_2_:Mn_3_O_4_ samples.

Sample	BET Surface Area (m^2^⋅g^−1^)	Pore Volume (cm^3^⋅g^−1^)	Pore Size (nm)	Average Grains Size (nm) ^(a)^
CeO_2_:Mn_3_O_4_ (7:3)	52	0.35	29	16
CeO_2_:Mn_3_O_4_ (3:7)	29	0.30	43	29

^(a)^ Using BET surface area.
